# Intravesical Oxybutynin for Urgent Bladder Rescue in a Newborn with Posterior Urethral Valves

**DOI:** 10.1055/s-0039-3399565

**Published:** 2019-11-22

**Authors:** Ada Molina Caballero, Alberto Pérez Martínez, Concepción Goñi Orayen, Gemma Sierra Colomina, Ana Lavilla Oiz, Yolanda Armendariz Cuevas

**Affiliations:** 1 Department of Pediatric Surgery, Complejo Hospitalario de Navarra, Pamplona, Spain; 2 Division of Neonatology, Complejo Hospitalario de Navarra, Pamplona, Spain

**Keywords:** posterior urethral valves, detrusor hypertrophy, oxybutynin, intravesical

## Abstract

Posterior urethral valves are the most common cause of bladder outlet obstruction in male newborns. Initial catheter drainage alleviates the urethral obstruction before definitive management by valve ablation. Newborns with posterior urethral valves often present with hypercontractile bladders that may inhibit upper tract drainage despite bladder catheterization. Anticholinergic agents are commonly used to treat detrusor hyperactivity, with oxybutynin being the most commonly used. We report the first case of a newborn with posterior urethral valves and ureterovesical junction obstruction caused by detrusor hypertrophy who underwent urgent intravesical instillation of oxybutynin at high doses in an attempt to avoid a diversion procedure.

## Introduction

Posterior urethral valves (PUVs) are the most common cause of bladder outlet obstruction in male newborns. Initial catheter drainage alleviates the urethral obstruction prior to undergoing valve ablation. Newborns with PUVs often have small hypertonic bladders with reduced capacity and increased detrusor contractility. 1 This hypercontractile bladder can inhibit upper tract drainage despite bladder catheterization.

Anticholinergic agents are commonly used to treat decreased bladder compliance and detrusor hyperactivity, with oxybutynin being most commonly used for patients with neurogenic bladder. 1 2 3 Its effectiveness is attributed to a combination of anticholinergic, antispasmodic, local anesthetic, and calcium-channel-blocking activities. 2 3 4 Long-term experience supports its safety in newborns and infants. 5 6 In children with insufficient response or significant systemic adverse side effects (ASEs) to oral oxybutynin, who would otherwise require surgical treatment, intravesical instillation of oxybutynin has emerged as an alternative in managing bladder dysfunction. This method has been found to be highly effective, reliable, and well tolerated. 3 4 7 8

We report the first case, to our knowledge, of a newborn with PUVs and ureterovesical junction obstruction caused by detrusor hypertrophy who underwent urgent intravesical instillation of oxybutynin at high doses in an attempt to avoid a diversion procedure.

## Case Report

**Fig. 1:**
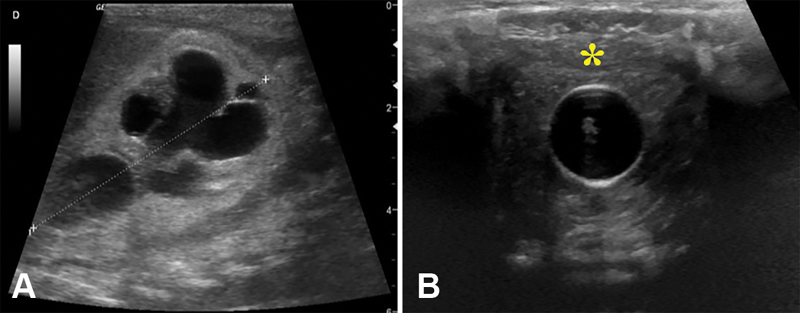
Ultrasound scan showing bilateral ureterohydronephrosis (A) and an empty bladder with the hypertrophied wall (asterisk) closely clasping the balloon of the vesical catheter (B).

**Fig. 2:**
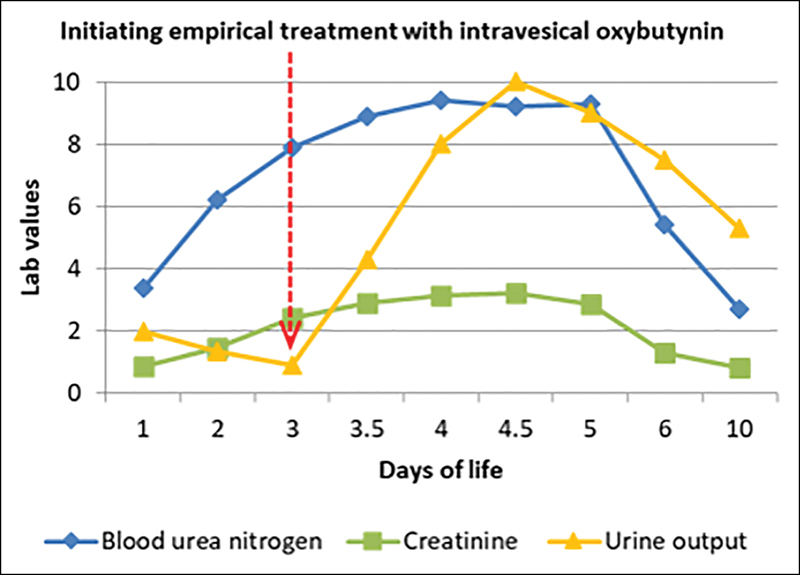
Graph showing increased urine output following the initiation of intravesical treatment with oxybutynin (red arrow), with complete correction of electrolyte disorders following 3 days of therapy.

The patient was a male newborn (birth weight: 2,270 g) with PUVs, bilateral ureterohydronephrosis, and significant bladder wall thickening. A Foley catheter placed at birth stopped producing urine at 12 hours of life. During the following 3 days, bilateral grade V ureterohydronephrosis and renal insufficiency with oligoanuria and severe electrolyte disturbances (hyponatremia, hyperkalemia, and a 3-mg/dL rise in creatinine) developed despite attempts to correct water and electrolyte imbalances by intravenous fluid therapy. On ultrasound, the bladder was empty, with the hypertrophied wall closely clasping the balloon of the Foley catheter ( Fig. 1 ). Prior to performing a necessary diversion procedure, we decided to try empirical treatment with intravesical instillation of oxybutynin (solution: 5 mg of oxybutynin hydrochloride and 20 mL of saline, instilling 4 mL every 4 hours = 2.6 mg/kg/day). With each instillation, the drug remained in the bladder for 15 minutes. Twelve hours following the initiation of treatment, urine output was 10 mL/kg/hour, with complete correction of electrolyte disorders within 3 days ( Fig. 2 ). No local or systemic ASEs were noted.

On the 15th day of life, endoscopic valve ablation was performed and we transitioned to oral oxybutynin (0.3 mg/kg/day). Oxybutynin was administrated for 18 months, with urodynamic studies showing an increased bladder capacity with low maximum filling pressures.

## Discussion

In 1989, Brendler et al first reported the use of intravesical oxybutynin (IVO) for the treatment of neurogenic bladder. 9 Since then, multiple reports of the efficacy of IVO have been published. 3 4 8 10 11 Treatment with oral oxybutynin has a high incidence of systemic anticholinergic ASEs. In some cases, adequate suppression of the detrusor can only be achieved when elevating the dosage, which will also increase the side effects. 2 IVO reduces first-pass metabolism in the liver, resulting in decreased generation of the N-desethyl metabolite, which is associated with the anticholinergic effects. 2 3 12 In addition, a pharmacokinetic study by Di Stasi et al reported a direct local effect of IVO within the bladder wall, probably through urothelial accumulation, suppression of muscarinic receptor-mediated detrusor muscle contractions, and blocking of muscarinic receptors in bladder-afferent pathways. 13 These pharmacological properties allowed us to delivered high doses of the drug to the target tissue with maximum pharmacological response and no ASEs.

Although the optimum dose for IVO has not been determined, a systematic review by Guerra et al states that most published studies used a dose of 0.2 mg/kg/day (average 10 mg daily). 3 Given the better tolerability and more rapid onset of action of IVO compared with oral treatment noticed in our experience with neurogenic bladder, we decided to further increase the dose in our patient. It seems that despite early relief of the bladder outlet obstruction, pathological changes in the detrusor from in utero obstruction resulted in an ureterovesical junction entrapment caused by an overactive hypertrophied detrusor. This was sufficiently intense to induce renal failure. Therefore, we needed a strong and fast detrusor-suppressing effect.

As myogenic failure is a legitimate concern when initiating any anticholinergic therapy, urodynamic monitoring is mandatory during treatment. However, drug-induced myogenic failure is reversible upon stopping the medication. 1 5 In addition, although our patient did not develop any ASEs with intravesical therapy, adverse effects are still possible, likely secondary to drug absorption through the bladder mucosa. 3

## Conclusion

This case suggests that an overactive hypertrophied detrusor contributed to the ureterovesical junction obstruction. It was reasonable to attempt to use an anticholinergic to adequately suppress detrusor activity. It was only the intravesical therapy that allowed high doses of oxybutynin to be administered with no side effects. This type of treatment should be considered as an alternative to an immediate diversion procedure in patients with PUVs. Nevertheless, the perceived positive effect of this treatment modality requires validation in controlled trials.

## Notes

New Insights & the Importance for the Pediatric Surgeon First reported use of intravesical oxybutynin as a measure to avoid a diversion procedure in a newborn with posterior urethral valves and ureterovesical junction obstruction caused by detrusor hypertrophy.
